# Analytic Programming with fMRI Data: A Quick-Start Guide for Statisticians Using R

**DOI:** 10.1371/journal.pone.0089470

**Published:** 2014-02-28

**Authors:** Ani Eloyan, Shanshan Li, John Muschelli, Jim J. Pekar, Stewart H. Mostofsky, Brian S. Caffo

**Affiliations:** 1 Department of Biostatistics, Bloomberg School of Public Health, Johns Hopkins University, Baltimore, Maryland, United States of America; 2 Department of Biostatistics, Indiana University Fairbanks School of Public Health, Indianapolis, Indiana, United States of America; 3 F. M. Kirby Research Center for Functional Brain Imaging, Kennedy Krieger Institute, Baltimore, Maryland, United States of America; 4 Department of Radiology, The Johns Hopkins School of Medicine, Baltimore, Maryland, United States of America; 5 Department of Neurology and Psychiatry, The Johns Hopkins School of Medicine, Baltimore, Maryland, United States of America; University of Minnesota, United States of America

## Abstract

Functional magnetic resonance imaging (fMRI) is a thriving field that plays an important role in medical imaging analysis, biological and neuroscience research and practice. This manuscript gives a didactic introduction to the statistical analysis of fMRI data using the R project, along with the relevant R code. The goal is to give statisticians who would like to pursue research in this area a quick tutorial for programming with fMRI data. References of relevant packages and papers are provided for those interested in more advanced analysis.

## Introduction

This primer was created to give statisticians who are new to the field of imaging data analysis a quick overview of functional magnetic resonance imaging (fMRI) data and to describe tools for programming their own analyses of fMRI. It is targeted at those who would like to pursue programming using the R project [1]. The key benefit of programming on one's own is the ability to extend analyses and build and create new tools. Of course, the key benefits of using the R project are that it is open source, cross-platform, free (as in cost), and is an award-winning lingua franca of statisticians. Matlab (www.mathworks.com) is the standard scripting language in neuroimaging and signal processing, and has a widely used integrated development environment and GUI creation software. Both R and Matlab are cross-platform and have excellent graphics capabilities. Both are high level scripting languages that are easy to learn and have a large collection of add-ons and subroutines.

For those wanting more automated methods for the analysis of fMRI data, several programs exist to perform most standard analyses. In addition, power users should learn these programs for comparison purposes to avoid reinventing existing and well established tools. We give a nonexhaustive list of some of our favorite freely available software below.

**SPM** SPM (http://www.fil.ion.ucl.ac.uk/spm/) is a collection of open source Matlab scripts, often calling compiled code. SPM is arguably the most popular software for the analysis of fMRI data, in no small part due to a well developed GUI. It has methods for single-subject and multi-subject analyses and tools for displaying the results and data visualization. It can perform all of the basic preprocessing procedures of the imaging data. Moreover, it has an active user community with several user contributed add-ons. The SPM scripts are well documented, and are easily understood.

**FSL** FSL (http://www.fmrib.ox.ac.uk/fsl/) is a UNIX-based software that performs single- and multi-subject analyses, preprocessing and visualization of results. It has a graphical user interface, though is most effectively used at the command line, tying routines together using shell scripts. FSL is open source and can be compiled from scratch. In addition, pre-compiled binaries are available, notably for OSX. For Windows, FSL can be run in a virtual machine.

**MIPAV** MIPAV (http://mipav.cit.nih.gov/) is a JAVA software created by the US National Institutes of Health. It has very broad functionality for preprocessing, analysis and visualization of imaging data. However, it is most useful through an active collection of modules.

**AFNI** AFNI (http://afni.nimh.nih.gov/afni/) is a UNIX-based software. It offers a full analysis and processing suite. We often use it in conjunction with FSL and BASH scripting.

**FreeSurfer** FreeSurfer (http://surfer.nmr.mgh.harvard.edu/) is a UNIX-based software for registration (often atlas based) and analysis of medical imaging data. It is often discussed as having excellent registration and visualization tools.

**ANTS** ANTS [3] is a UNIX-based software for preprocessing of imaging data. It provides methods for linear and nonlinear registration of images.

In addition to the above tools for analysis and processing of fMRI data, there are R packages related to fMRI. We list a few packages here primarily focused on analysis postregistration.

**AnalyzeFMRI** [3] Analysis package for reading and writing of fMRI including a graphical user interface. It can also be used for performing temporal and spatial ICA.

**oro.nifti** [4] Package for reading in NIfTI files, a common fMRI imaging format. It can be used for reading compressed files.

**oro.dicom** [5] Package for reading in DICOM files, another common fMRI imaging format.

**fmri** [6] Package for reading in and analyzing fMRI data. Includes graphical user interface for fMRI analysis and adaptive smoothing for fMRI along with inference.

**neuRosim** [7] Package for simulating fMRI data. As a consequence, has tools for creating and investigating designs with HRF convolution models.

**arf3DS4** [8] Package for activated region fitting for fMRI data in NIfTI format.

**Rniftilib** [9] Package for loading and writing the 3D or 4D images.

**RNiftyReg** [10] Package for 2D or 3D image registration.

**brainwaver** [11] Package for functional connectivity analysis.

**tractor.base** [12] Package for reading, writing, and graphical representation of images.

**waveslim** [13] Package for wavelet analysis of 1D, 2D and 3D images.

**cudaBayesreg** [14] Package for Compute Unified Device Architecture (CUDA) based Bayesian multilevel analysis of fMRI data.

We will use some of the packages above in the examples in this manuscript. In addition, a frequently updated more exhaustive list on CRAN of packages for medical image analysis can be found here:

http://cran.r-project.org/web/views/MedicalImaging.html.

The remainder of the manuscript is organized as follows. Sections 1, 2 and 3 describe the structure of the fMRI data, discuss ways of obtaining the data and give a brief overview of the preprocessing steps. Section 4 describes the different formats that can be used for viewing the fMRI data in R along with examples of obtaining a Gaussian smoother of the data matrix and the mask of the three dimensional image. A discussion of the hemodynamic response (HR) function and the role of the design matrix in task-based fMRI studies is presented in Section 5. The between-subject random effect models are discussed in Section 6. Section 7 gives an overview of the connectivity based analysis of fMRI data including methods based on seed-voxel analysis, singular value decomposition and independent component analysis. As the main goal of the manuscript is centered on the statistical analysis of fMRI data via R, we do not present visualization tools in detail, however, we briefly discuss a few of the visualization tools in R or other software in Section 8. Finally, the last section completes the article by pointing out useful further reading material.

## Materials and Methods

### 1 fMRI

FMRI is a modern brain imaging measurement technology. As its name suggests, the technology is used to explore brain function (activity) by obtaining several images of the brain over time using an MRI machine. Standard fMRI images the so-called blood oxygen level dependent (BOLD) signal, described further below. An extensive overview of the statistical methods developed for the analysis of fMRI data is presented by [15], [16], [17] and [18]. Herein, we will discuss the implementation of some of these commonly used techniques in R, while providing brief discussions as to the methods and the interpretation of the results. BOLD fMRI is not the only functional brain imaging technology available using an MRI scanner (see [19] for an overview). For example, arterial spin labeling [20] is a closely related functional MRI technology. However, the term fMRI, when used without qualifying statements, refers to BOLD fMRI. We note that positron emissions tomography (PET), single photon emissions computed tomography, electroencephalograms (EEG), magnetoencephalograms (MEG) and other non-magnetic resonance based measurement devices can be used for functional brain imaging, each with different goals, limitations, strengths and scientific interpretations.

We will not thoroughly discuss the details of the methods by which the MRI scanners use the principles of magnetic resonance to achieve different images. However, newcomers to the area may be surprised to find the amazing variety of biological signals that one can visualize using MRI. These include separate modalities to study white matter, gray matter, overall structure, white matter tracts, tracers injected into the body and lesions in the brain. There are multiple types of structural and functional images, all acquired using the principles of MR. See [19] and [21] for a general introduction to MR imaging.

**Figure 1:**
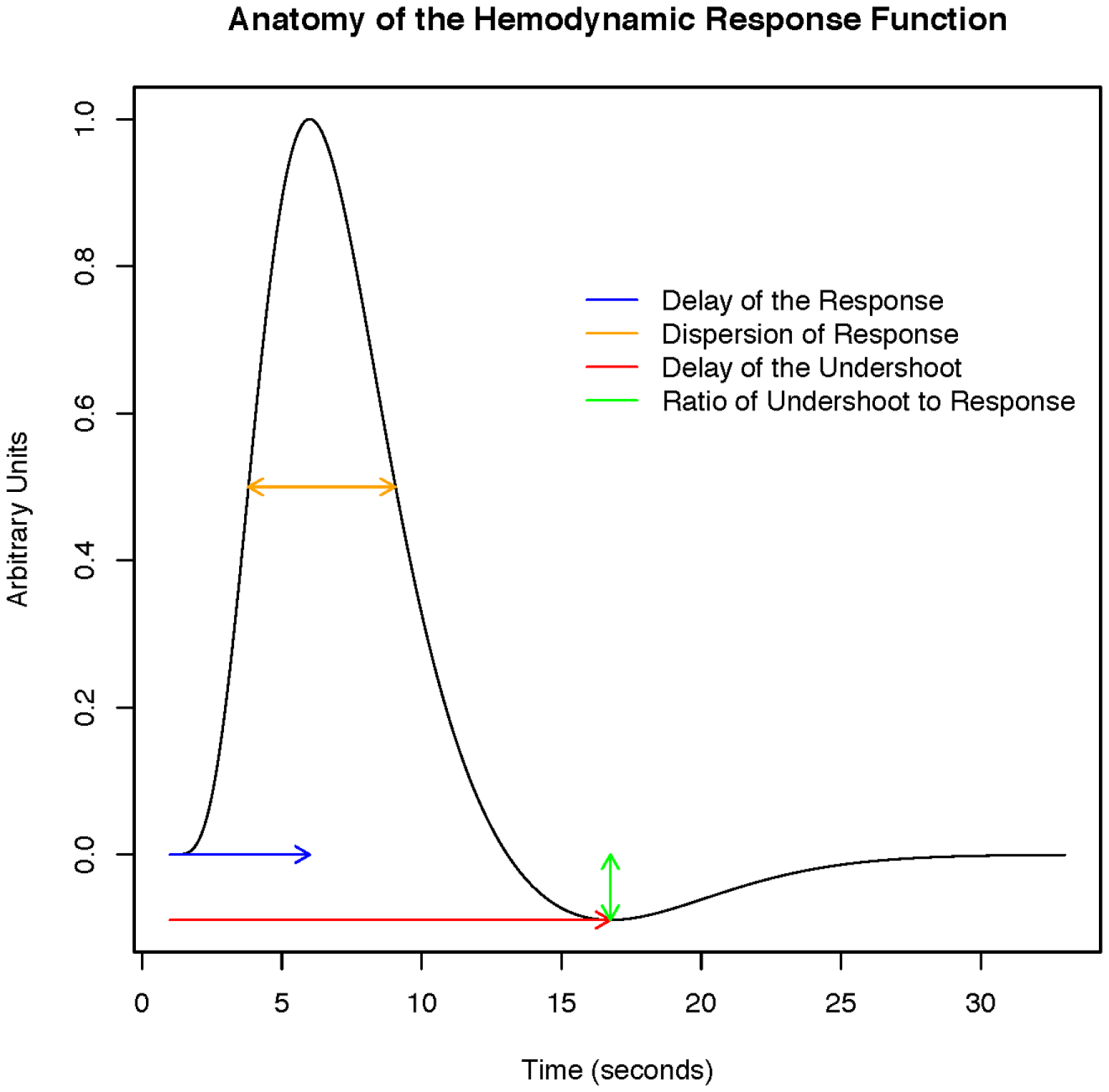
Anatomy of the HRF.

The BOLD signal ([22], [23]) measures the cerebral hemodynamic changes concomitant to neuronal activation of the brain. A localized increase in neural activity results in an increase in blood flow (“reactive hyperemia” [24],[25]) to the activated region when excess of oxygenated hemoglobin is delivered to the region. Consequently, a reduction in deoxyhemoglobin concentration follows, resulting in an increase in magnetic field homogeneity. The gradient echo EPI sequence ([26], [27]) allows to study the activation of specific regions of the brain during a task. Deoxyhemoglobin serves as an endogenous susceptibility contrast agent allowing MRI to report on local hemodynamic changes. Hence, the BOLD signal in response to a stimulus is given a name - the hemodynamic response. Figure 1 shows an example hemodynamic response function (HRF). In Figure 1, an initial delay of the response can be observed since it takes time for the vasculature of the brain to respond to the need for oxygen after the stimulus. This is followed by a subsequent brief undershoot [28]. The origin of the undershoot is controversial. One explanation of the undershoot is that there is ongoing oxygen consumption after reaching the point of origin. The other is that the excess volume of deoxygenated blood results in delayed vascular compliance.

In BOLD fMRI the scanner records images approximately every second (the so-called TR or time of repetition). At each time point, a 3D image of the brain is obtained. Often, in BOLD fMRI, the image is collected one slice at a time for each time point instead of scanning the 3D image immediately. Hence, a slice time correction (e.g., by Fourier transformation [28]) is applied to the resulting images as part of the preprocessing. Empirical face validity of the methodology can be demonstrated by simple motor or visual tasks. For example, consider a “finger tapping” task where a subject is asked to tap their finger while in the scanner and then rest, repeating this sequence for a certain time interval. When the images are averaged over the times at rest and compared to the times when tapping the finger, and appropriate statistical tests are performed, motor areas of the brain are clearly activated (see [29] for overview of finger tapping task-related analysis in the literature). By virtue of the well-defined motor area of activation and ease of performing the task, finger tapping tasks are commonly used for scanner and methodological validation. They are also of intrinsic interest in the study of motor areas. Various other tasks can be designed depending on the goals of the study. More recently, resting state fMRI has been used to explore the brain function when the subject is at rest. Section 5.1 provides details on how the experimental design can inform the statistical analysis of the data.

The development of the BOLD fMRI technology facilitated research in the analysis of cognitive function of the brain [15]. For instance, one may be interested in identifying which regions are involved in performing a mental operation or a cognitive task, how active is a brain region during a task, what is the shape of the time course of activation or quantifying the extent of connectivity between brain regions during tasks. Since there are several sources of variability introduced during data collection, statistical methods are required in order to accurately obtain the regions of interest. For instance, in Section 5.1 we discuss two types of experimental designs used frequently in fMRI analysis: block design and event-related design. It can be shown that the block designs have high detection power (i.e., the ability of the method to detect activation), whereas the event related designs have better estimation efficiency in that they can be used to estimate the shape of the HRF [30]. In Section 5.1, we show several statistical methods that can be used to answer the questions posed above.

**Figure 2:**
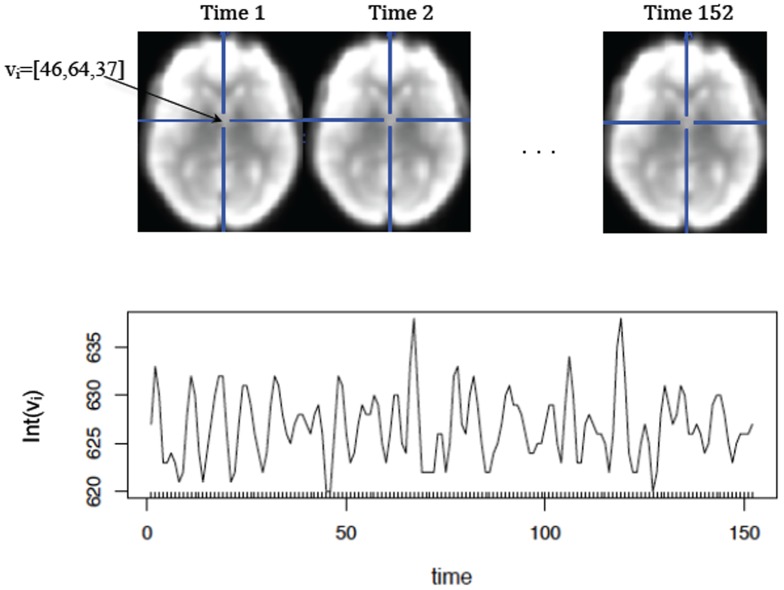
Resting state fMRI image for 1 subject. The top panel shows one axial slice from the 3D image acquared at each of the 152 time points. 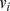 denotes a voxel in the brain with coordinates [46, 64, 37]. The plot below shows the intensity of the image at voxel 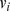 (on the y-axis) at each time point (the x-axis corresponds to time).

For the purpose of statistical analysis the observed fMRI dataset contains a 3D array of intensities for each time point. The number of timepoints can vary depending on the length of the scan and is usually in hundreds. Axial slices of the 3D images at each time point for a subject at rest are shown in Figure 2 top panel. A voxel (or volumetric pixel) is defined as a 3D unit element in the image. The background of the image is generally removed and the voxels in the brain are used for analysis. Depending on the research question one may choose to organize the data differently. For instance, the time courses of each voxel (as shown in Figure 2 bottom panel) may be analyzed (voxel-wise) to identify the voxels that are activated during a task. The 3D images at each time point may be vectorized and concatenated to obtain a 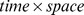 matrix that can be further analyzed to discover brain networks. The correlations of time courses for each pair of voxels in the brain are often used in connectivity analysis, that is in identifying areas of the brain that activate and deactivate together.

### 2 Obtaining data

As many neuroscience researchers are joining open science initiatives promoting public access for data, numerous websites offer datasets that can be downloaded freely. However, we have a few favorite canonical datasets that are good starting points for analysis. The first are SPM's example datasets that can be used for single-subject and inter-subject analyses. The second dataset of interest is the 1000 Functional Connectomes Project (FCP)/INDI [31] resting-state data. Most of the SPM datasets are preprocessed and ready for analysis; however, the FCP data need extensive preprocessing. UNIX shell scripts using FSL and AFNI can be found on their website for those who are interested in preprocessing.

The SPM example datasets can be downloaded at

http://www.fil.ion.ucl.ac.uk/spm/data/

The 1,000 FCP dataset can be downloaded at

http://www.nitrc.org/projects/fcon_1000/

As mentioned above, the 1,000 FCP data involves the most preprocessing. We therefore put a processed image in .nii format along with the corresponding 2D matrix in an R data file format with the extension .rda (the .rda files can be loaded by using the load() function in R) for download on our servers at

www.biostat.jhsph.edu/~bcaffo/downloads/rest_res2standard.nii.gz www.biostat.jhsph.edu/~bcaffo/downloads/imageDataMatrix.rda

For illustrating the analysis in this manuscript, we choose images from the Kennedy Kreiger Institute (the home institution of some of the coauthors of this manuscript).

We also note that FSL has an evaluation suite, the FEEDS dataset, that we have found very useful, both for learning FSL as well as debugging new methods. The FSL example dataset can be downloaded at:

http://www.fmrib.ox.ac.uk/fsl/feeds/doc/index.html

### 3 Preprocessing

Preprocessing is an essential component in the analysis of fMRI data. Standard preprocessing requires, for example, skull stripping, evaluation and corrections for motion, coregistering, spatial smoothing and registration to a template. We discuss two preprocessing steps in particular: spatial smoothing and registration to a template. However, for newcomers to the area, we suggest leaving all the steps, except for possibly the spatial smoothing to standard software, or acquiring data where preprocessing has already been completed in an acceptable manner. For those readers interested in pursuing preprocessing as a research endeavor, good theoretical foundations are given by [32], [33] and [34].

Warping the images of a subject to a template, often called spatial registration or spatial normalization, is the key step of attempting to put subject images into a common space. That is, each registered image is transformed to the same space and hence the spatial locations of the voxels in the brain are ideally interpretable across subjects, in other words, voxel 1 for subject 1 is the same as voxel 1 for subject 2. In functional neuroimaging, this process relies on numerous assumptions. For example, it presumes anatomical similarity across subjects at the functional resolution of interest. This assumption becomes especially problematic when studying diseased brains, where anatomy can be drastically different than the healthy control template brain. Also, it presumes localization of the brain function of interest to common anatomical locales across subjects.

Setting these issues aside, registration is accomplished by mathematically warping each subject's collection of images over time to a so-called “template”. A template is typically a highly detailed structural image obtained by imaging a subject repeatedly or warping several subjects into one common image. The template is useful for overlaying results on top of it which then helps in contextualizing the results. It is important to know what template was used to warp the images one is analyzing. Because it is often a highly accurate structural image (or there is an associated image in the same space), it is useful for interpretation, as neuroscientists can visually relate findings to important structural landmarks. Moreover, some templates are in wide use and key regions have been tabulated (see [35] for more details). We normally use templates created by the Montreal Neurological Institute (MNI) at:

http://imaging.mrc-cbu.cam.ac.uk/imaging/MniTalairach

The International Consortium for Brain Mapping (http://www.loni.ucla.edu/ICBM/) releases labeled brain atlases. We should also mention the famous Talairach Atlas, which is a coordinate system created before MR imaging was in place. Relating results to Talairach coordinates is often done to put results in a historical perspective familiar to neuroscientists. Some templates have conversion utilities to convert their coordinates to Talairach coordinates.

Finally, spatial smoothing using a filter is a step that can be easily done in R. The choice of whether spatial smoothing is necessary to perform is generally a designed component of a statistician's analysis plan. Hence, of all the preprocessing steps, this is the one we suggest to be performed in R if deemed necessary.

### 4 Loading data and structures

#### 4.1 Array format

Two key data representations are the array format and the matrix format. The first is the more natural format for the data. Suppose I is an array representing a 3D image of the brain, where I[i,j,k] is the intensity of voxel i,j,k. If the image is collected repeatedly over time (as in fMRI), then 4D arrays ( I[i,j,k,t]) are useful, though often are very large.

Medical images in neuroimaging are usually saved in one of a few available formats. However, all formats have common properties, for instance, they are binary and the data are stored as a long floating point vector. Header information includes descriptive features of the image such as the physical dimensions of a voxel and the size of each of the three dimensions, as well as other information.

**DICOM** Digital Imaging and Communications in Medicine (DICOM) is a standard image format from National Electrical Manufacturers Association often used as scanner output. DICOM images have the header information built into the binary file. 3D DICOM files are sometimes split into several files of 2D slices, but can be combined into a single file.

**Analyze** The Analyze format includes two files for each 3D image: a header file with the extension .hdr and an image file with the extension .img. The header and image files have to have the same filename and have to be saved in the same folder. The 4D fMRI images are often stored as separate files, where the 3D image for each time point is saved as a separate file. In Analyze 7.5 format, the 4D images can be saved using one .img and one .hdr file.

**NIfTI** NIfTI files typically have the extension .nii and are a current standard for the analysis of fMRI data. NIfTI files can have more than 3 dimensions. Often NIfTI files are compressed using gzip and so the file extension is .nii.gz. If your analysis program cannot gunzip the file, simply uncompress it using any of the standard compression software tools and the result will be a standard (uncompressed) NIfTI file. The R package oro.nifti can be used to read in the gzipped files directly.

In this example, we read in a sample 3D so-called contrast image and smooth it using a 3D Gaussian filter [36] included in the package AnalyzeFMRI. For illustration, we are using one of the SPM datasets (see [37], for more information about the dataset) described in Section 2. First we read in the data.

library(AnalyzeFMRI)

imageFileName <- “pathToImageFile/imageFile.img”

img <- f.read.analyze.volume(imageFileName)[,,,1]

Here we assume that imageFile.img is an Analyze image file with a header and image pair: imageFile.hdr and imageFile.img. Notice this data is 3D, yet the program reads it in as 4D, where the first three dimensions correspond to the size of the 3D image and the fourth dimension is set to 1. Hence the array subscripting drops the dimension by 1. It is often useful to read in the header information.

headerFileName <- “pathToImageFile/imageFile.hdr”

hdr <- f.read.analyze.header(headerFileName)

We can visualize this image using the image function.

# visualize the image

tempImg <- img

tempImg[is.na(tempImg)] <- min(img, na.rm  =  TRUE)

for(k in 1: dim(img)[3])

image(tempImg[,,k], axes  =  FALSE)

Note that the creation of the temporary image tempImg in this example is only useful as the background of the raw image is stored as NA. Note also by default the image command does not scale the image relative to the actual voxel dimensions. The parameter asp can be used to specify the aspect ratio y/x which would result in the correct voxel dimensions. The following code smooths the image using the function GaussSmoothArray.

simg <- GaussSmoothArray(tempImg,

voxdim  = c(3, 3, 3),

ksize  = 5,

sigma  =  diag(4, 3),

mask  =  as.integer(!is.na(img)))

for(k in 1: dim(img)[3])

image(simg[,,k], axes  =  FALSE)

**Figure 3:**
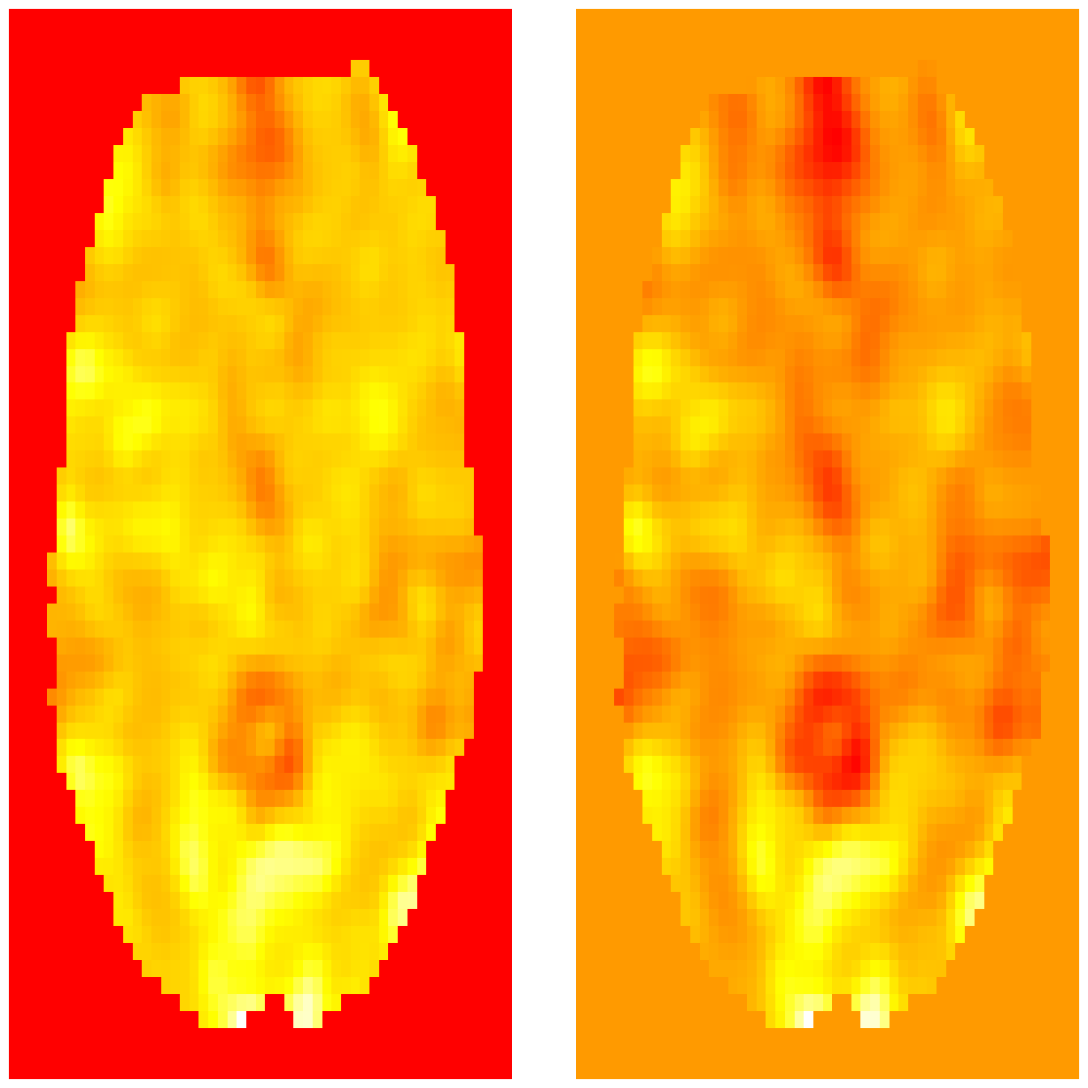
A slice of the fMRI image plotted by using the image() function in R (left) along with the corresponding smoothed slice (right). Here the red color corresponds to high intensity values, followed by yellow and white as the values decrease.

Here the mask statement contains the smoother to the non-background areas, i.e., the smoother is applied to the non-background voxels which have the value of 1 in the mask. The voxdim variable contains the voxel dimensions, in this case three millimeters cubed. The voxel dimensions can be found using visualization programs or inspecting the hdr variable. The ksize and sigma parameters are smoothing parameters. The sigma parameter is the standard deviation of the Gaussian kernel (specified in mm) while ksize is the truncation width. See the documentation for GaussSmoothArray for more information. Figure 3 shows an axial slice of the original image along with the corresponding smoothed image (see Section 8 for more details on displaying images).

#### 4.2 Matrix format

The array format, while useful for smoothing, is not a convenient data structure for most other analyses. Most importantly, it is not a parsimonious structure, since it includes all of the voxels outside of the brain, i.e., the background of the image. In some fMRI analysis, where the spatial structure of the voxels is disregarded, a common **mask** is applied to the 3D data and the data are then saved in a long 1D vector. In fMRI, if there is one vector per time or subject, these can be stacked into a matrix for analysis. Under this structure, any analysis of the resulting matrix must be invariant to spatial location. The spatial location information can be kept elsewhere for back reconstruction of the image. For example, if there are 30,000 nonbackground voxels, one could retain the 3
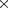
30,000 matrix of location indices for those voxels. Masks can be created in a variety of ways. They can be obtained from the template or from the data itself. The mask is an important structure to retain, as it can map a vector back to an array format for display.

Here, we will take a collection of 3D images for one subject (one 3D image per time point) and create a mask. Using the mask, we will then create the data matrix. First, we read in the list of the files (with absolute paths).

library(AnalyzeFMRI)

# the directory where the images are stored

fileDir <- “pathToImageFile”

# the collection of images

files <- dir(fileDir, pattern  =  “*.img”, full.names =  TRUE)

It is useful to have the image dimensions assigned to an object. We are assuming that all images are registered and have the same dimensions.

# obtain the image dimensions by loading the first image

imageDim <- f.read.analyze.header(files[1])$dim[2: 4]

Now we loop over the time courses and collect them into a matrix by grabbing the relevant indices across the time courses. We then find the corresponding non-background indices and place them in a vector called mask.

mask3D <- array(1, imageDim)

for (file in files) {

img <- f.read.analyze.volume(file)[,,,1]

mask3D <- mask3D * (!is.na(img))

}

# the mask is a list of indices

mask <- which(mask3D  =  = 1)

Next, we loop over scans and collect them into a matrix by grabbing the relevant indices across scans.

# now cycle through the data and get the data into a matrix format

dataMatrix <- NULL

for (file in files) {

# load file and make 3D

img <- f.read.analyze.volume(file)[,,,1]

dataMatrix <- rbind(dataMatrix, img[mask])

}

noScans <- nrow(dataMatrix)

noVoxels <- ncol(dataMatrix)

### 5 Within subject paradigm analysis

#### 5.1 Discussion on designs

**Figure 4:**
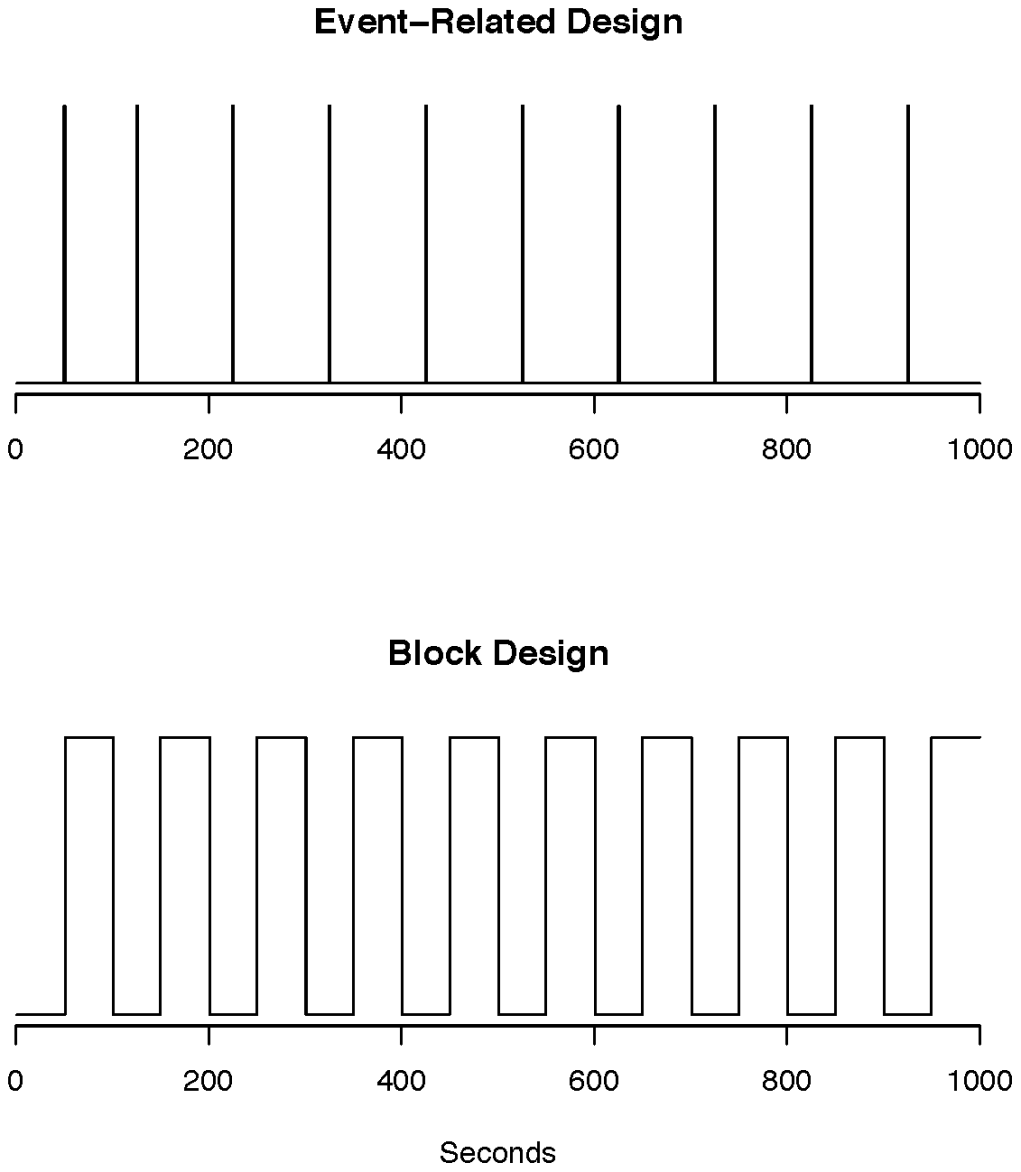
Stimulus vectors for an event-related design (top) and a block design (bottom). The spikes correspond to the onsets of stimuli. There is a sustained period of activity/task in the block design. In both cases, the spacings between events can be unequal.

When designing the experiment in task-based fMRI the aim is to maximize the statistical power in detecting activation during the task while assuring the validity of the psychological results. In task-based fMRI, there are two common designs: event-related and block design. Event-related refers to multiple stimuli that are assumed to occur instantaneously and have randomized time between events. A simple task would be to have the participant push a button approximately every 20 seconds to look at motor cortex activation though the events do not have to be equidistant. If a “continuous” task is performed, such as sequential finger tapping, for a certain time interval followed by a short block of rest, the design is referred to as block design. Examples of stimulus functions in each design are presented in Figure 4. The choice of event-related design versus a block design depends on the final goal of the experiment. The block designs lead to higher detection power, however, the subjects may learn the patterns of the experiment which may affect the interpretation of the results. Event-related designs reduce the effects of learning, boredom and other events unrelated to the task while exhibiting loss in detection power (see [17] for more discussion on designs and [38] on optimization of design parameters).

Suppose we have fMRI data for one subject during a task. The goal is to identify the area of the brain that activates during the task, which is performed by the subject. Typically, the fMRI data can be modeled as a sum of response, drift and noise:
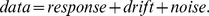



The response (i.e., the BOLD response) is often modeled using a linear time invariant system (see [17] for other ways of modeling the BOLD response). Assume the response is a linear combination of responses from 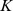 different stimuli. That is, the response for voxel 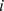, 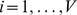 at time 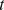, 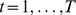, can be written as 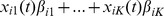, where 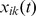 is the response from stimulus 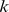 at time 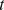 in voxel 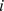, 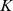 is the number of stimuli, 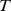 is the number of scans and 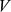 is the number of non-background voxels in each scan. The response from each stimulus depends on the stimulus function and the HRF, often modeled as a convolution of the two functions 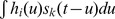, where 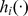 is the HRF at voxel 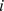 and 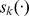 is the stimulus function corresponding to stimulus 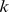. The modeling of the HRF function is an important part of the analysis and is discussed in more detail in Section 5.2.

In addition to the random variability, periodic noise may be present in fMRI data unrelated to the true BOLD response. This can result, for example, from the patient's heartbeat or other systematic effects. As a result, some voxels in the brain may show considerable drifts over time. The drift can be linear or nonlinear, hence a flexible polynomial model is often employed to allow for nonlinear effects in the drift. For example, a polynomial drift of order 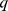 for scan 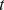 at voxel 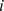 can be written as 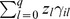, where 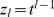. Finally, a random error 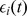 is added to the observed data, 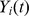, at voxel 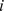 scan 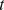 yielding
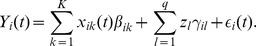



In Section 4.2, we discussed how to construct a matrix from the 4D fMRI array. The resulting matrix is 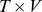 dimensional: the columns of which correspond to time courses in each voxel and the rows are the spacial images for each scan. We define the vectorized 3D image for each time point t as 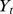 and
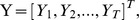


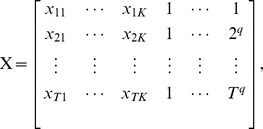
and
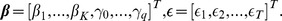



The general linear model (GLM) can be written in matrix notation as
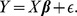



Note that, in the above equation, 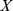 is derived based on specific hemodynamic assumptions, and 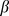 is interpreted as the size of the hemodynamic response (see [39] for more details). The random errors 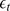 are generally not independent in time. The correlation structure can be specified based on assumptions one is willing to make about the dependence structure over time. Misspecification of the correlation structure in GLM can lead to biased estimation of coefficients. For example, the temporal correlations can be modeled using a first order autoregressive model [40], that is, 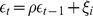, where 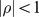 and 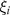 are independent and identically distributed. After estimating the parameters in the GLM model, the p-value maps of the 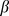 coefficients are thresholded to find the voxels where the corresponding 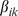 is significantly different from 0.

#### 5.2 HRF modeling

In Section 1 we discussed the HRF function, an example of which is shown in Figure 1. The estimation of the HRF function is often of interest in fMRI analysis. The HRF model in SPM implies that the HRF is a discretized difference of two gamma functions (see [15], [40])
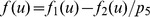


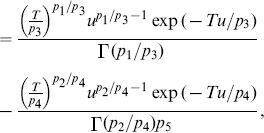



for 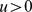. Typically, it is also normalized, either divided by its integral or maximum, to have an average or peak value of 1 respectively. The logic behind this specification of the HRF is that the initial post-stimulus increase in blood oxygenation is represented by 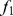, with the subsequent depletion represented by 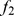.

Notice that 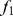 is a gamma density with shape 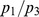 and rate 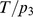, and 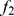 is a gamma density with shape 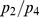 and rate 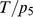. The SPM documentation defines 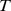 as the TR, 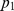 as the delay of the response to the onset, 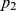 as the delay of the undershoot relative to the onset, 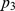 as the dispersion of the response, 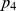 as the dispersion of the undershoot and 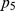 as the ratio of the response to the undershoot. Conceptually, these are illustrated in Figure 1. We briefly give motivation for these parameters. Note, the mean of density 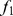 is 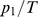 while the mean of density 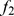 is 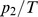. Hence, if 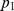 and 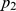 are given in seconds and 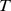 is seconds per scan, 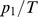 can be thought of as the delay to the mean (not modal) response in TR units while 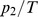 is the delay to the mean undershoot in TR units. Given that the gamma variance is 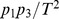, and the mean is 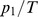, 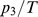 is the factor by which the response gamma distribution mean is scaled to obtain its variance. Similarly, 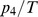 is the factor by which the undershoot gamma distribution mean is scaled to obtain its variance. Since the integral of 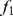 and 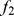 are 1, 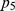 is the ratio of the two functions comprising the HRF. Note, it is not, as its name suggests, the ratio of the peak value of 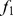 to the peak value of 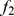.

The following example illustrates the use of the fmri.stimulus function in R to create the expected HRF as described by [40] and [6]. More appropriate choices of an HRF, such as spatially varying HRF and dynamic sets of basis functions may also be used [41]. For instance, finite impulse response models and Fourier basis sets are more flexible alternatives to the canonical HRF.

The R package fmri can be used to analyze fMRI data from one subject and identify activation during a task. In the following example, suppose imageFile.nii is a NIfTI data file. We first extract the 4D numeric array using the function read.NIFTI. The onset times of the stimulus (5.25, 21.45, 100.12, 223.5) and the durations of ON stimuli in scans (5, 5, 10, 10) are known from the experimental design. Next, we create the expected response for each stimulus (defined by 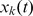 above, assuming that the HRF is the same in each voxel) using the function fmri.stimulus. The function fmri.design is invoked to create the design matrix. Finally, the coefficients of the hemodynamic response to the stimulus are evaluated in the GLM [42].

library(fmri)

imageFileName <- “pathToImageFile/imageFile.nii”

img <- read.NIFTI(imageFileName) onsets <- c(5.25, 21.45, 100.12, 223.5)

dur <- c(5, 5, 10, 10)

hrf <- fmri.stimulus(scans  =  img$dim0[4],

onsets  =  onsets,

durations  =  dur)

x <- fmri.design(hrf, order  = 2)

For event-related designs, the durations of the events would be considered instantaneous. Hence, the duration in the function fmri.stimulus would be 0. The example above is an example of a block design. The argument order in the function fmri.design is the order of the polynomial drift term of the design matrix, the default value of the order of the polynomial is 2. If there are 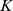 experimental stimuli, there will be 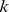 expected BOLD responses 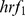, 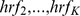. Note that in this example 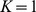. The design matrix can be created as follows

x <- fmri.design(cbind(hrf_1, …, hrf_K), order  = 2)

Given the design matrix, we may conduct voxel-wise analysis of the brain using regression models to identify the areas activated during the task. Suppose 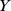 is the 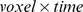 matrix. Then we can use the following code for the analysis with the design matrix above via the fmri.lm function in fmri package.

model <- fmri.lm(img, x, keep  =  “all”)

The argument keep in the function fmri.lm describes the parts of the output returned by the function. The default is keep = “all”, where residuals are included in the returned object. Alternatively, we can perform similar analysis via the lm function as follows. First, extract the data array and construct the data matrix as discussed in Section 4.2.

ttt <- extract.data(img)

mask <- img$mask

dataMatrix <- NULL

noScans <- img$dim[4]

for(t in 1:noScans) –

scan <- ttt[,,,t]

dataMatrix <- rbind(dataMatrix, scan[mask])

}

We can now run the lm function for each voxel and extract the coefficients and p-values. Note, we only use the lm function for didactic reasons, as the calculations can be done more efficiently using other approaches.

lms <- apply(dataMatrix, 2, function(y) lm(y∼x))

# obtain the summary statistics for each voxel

summaries <- lapply(lms, summary)

# Coefficients

coefs <- lapply(summaries, function(x) x$coefficients)

# Intercept map

int_vals <- sapply(coefs, function(x) x[“ (Intercept)”, ])

int_est <- int_vals[“Estimate”,]

int_pvals <- int_vals[“Pr(>|t|)”,]

# Slope map

beta_vals <- sapply( coefs, function(x) x[2,])

beta_est <- beta_vals[“Estimate”,]

beta_pvals <- beta_vals[“Pr(>|t|)”,]

As a result of fitting the linear model, we obtain coefficients and their p-values for each voxel. The resulting p-values can be thresholded using a multiple comparisons correction procedure. Finally, the map can be saved in an image format to visualize the areas that are significantly associated with the task.

### 6 Between-subjects random effect models

In the fMRI literature, “random effect analysis” refers to an inter-subject analysis of paradigm-related contrast images. Specifically, in the first stage, subject-specific regression models are fit, relating the HRF-convolved design matrix to the fMRI time series at each voxel separately. Coefficients or estimable linear combinations of coefficients (so-called contrasts) are obtained from the fit. For each subject, as there is one contrast per voxel (one coefficient per voxel), the resulting collection of voxel-specific contrast values is referred to as the subject specific contrast image, and is usually stored as a 3D image. Random effect models then analyze these images across subjects. That is, they compare inter-subject variation in estimated hemodynamic response to the paradigm to variation in covariates, diagnoses, treatments, and so on. The name “random effect analysis” is given as the process is an approximation of formal random effects.

#### 6.1 Example: group activation

Below, we consider a simple form of random effect analysis. We take the subject-specific contrasts and test whether their voxel specific mean is different from 0. One of the SPM datasets discussed in Section 4.1 is used to illustrate the results. We will assume that the following steps have been completed using the code provided in Section 4.2∶1) a mask containing nonbackground voxels has been created, 2) the mask has been applied to every subject's contrast map resulting in a vectorized map, 3) the data have been concatenated into a matrix containing noSubjects (number of subjects) rows and noVoxels (number of voxels) columns, 4) the data matrix is assigned to the variable dataMatrix. In our case, there are 12 subjects and roughly 50,000 non-background voxels. As a first test, consider a test of 0 mean applied at each voxel.

# apply the function t.test to every column (voxel)

ttest.out <- apply(dataMatrix, 2,

function(x) {

temp <- t.test(x)

c(temp$statistic, temp$p.value)})

# label the rows

rownames(ttest.out) <- c(“statistic”, “pvalue”)

**Figure 5:**
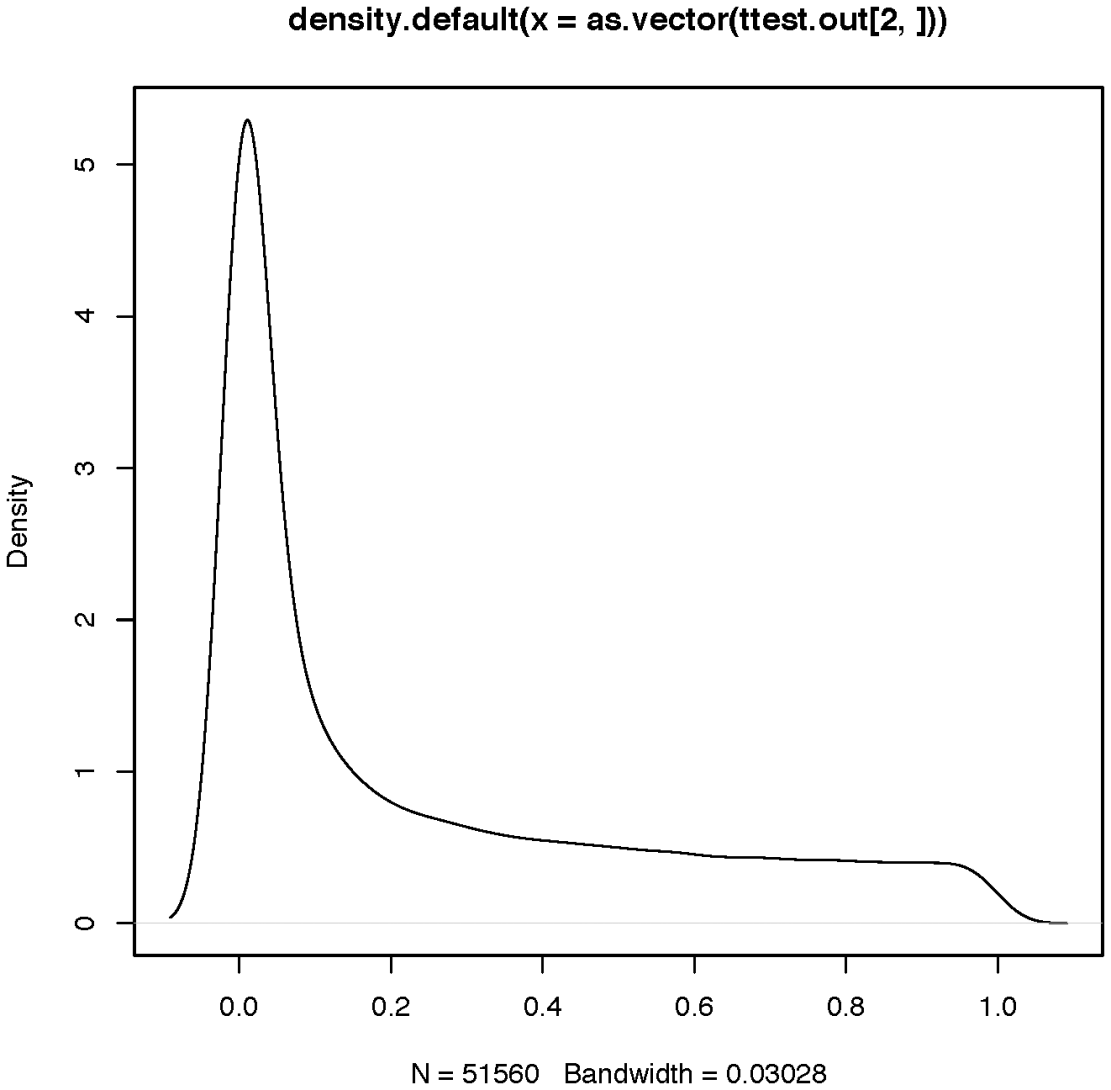
P-value histogram from a group-level test.

Figure 5 displays the p-value histogram. Next, we threshold these p-values using the false discovery rate (FDR) threshold in AnalyzeFMRI package.

Threshold <- Threshold.FDR(ttest.out[1,],

q  = .05,

cV.type  = 2,

type  =  “t”,

df1  =  noScans - 1)

**Figure 6:**
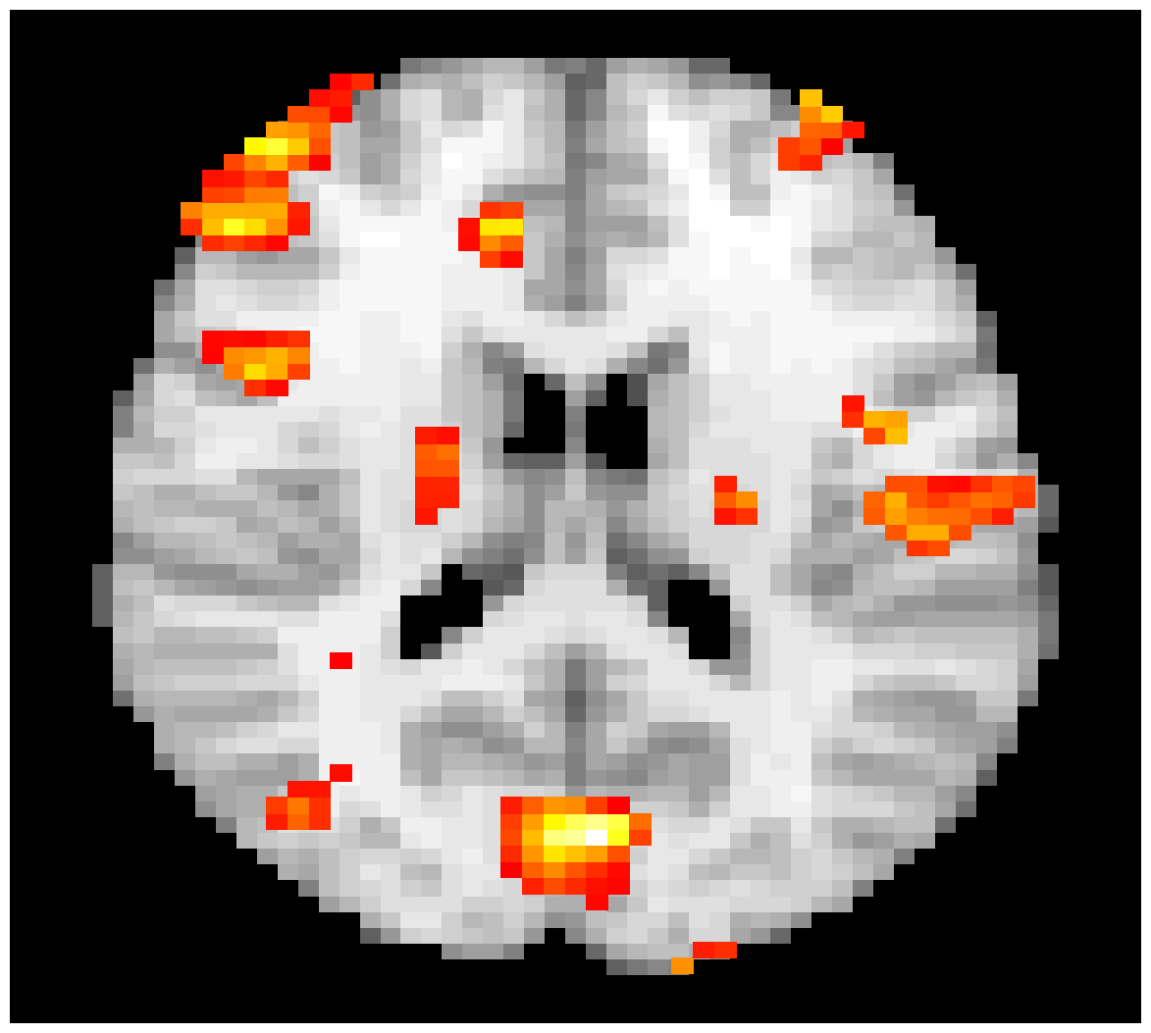
A slice of the thresholded output map overlaid on a template brain using the image() function.

Now we can create an image where we plot the thresholded p-values overlaid on a template brain. The resulting image can be plotted using the image function in R as described in Section 8. A slice from the resulting image overlaid on a template brain is shown in Figure 6.

outputImage <- array(0, imageDim)

toSave <- ttest.out[1,] > =  Threshold

outputImage[mask[toSave]] <- ttest.out[1, toSave]

#### 6.2 Example: permutation testing

Permutation testing [53] is often used to identify changes in activation in a group of subjects based on a covariate, such as disease diagnoses. As in example 6.1, suppose the mask has been created and the images have been vectorized and saved in the matrix dataMatrix. In addition, each subject's disease status is given as 0 for control or 1 for disease group. For any non-background voxel, if there is no difference in the experimental effect between the control and disease groups, then the labeling of each subject as 0 or 1 should be arbitrary. The labels can be permuted to obtain the t-statistic (e.g., mean difference between the two groups) for that voxel corresponding to each permutation. The distribution of the resulting test statistics for all permutations is often referred to as the permutation distribution.

# Number of subjects

n.s <- 12

n<- 2∧n.s

# Matrix of all possible combinations of 1 and 0 for each subject

perm.mat <- matrix(1,n.s,n)

for(i in 1:n.s){

p<- n/(2∧i)

j<- 1

while(j<(2∧i)){

perm.mat[i,(p*j+1):(p*(j+1))] <- 0

j<- j+2

}}

The R function expand.grid() can also be used to obtain the grid for the permutation test above. For illustration purposes, we compute permutation distributions for two randomly selected voxels.

# Randomly select two voxels

perm.noVoxels  = 2

sub.dataMatrix  =  dataMatrix[,sample(noVoxels, size  =  perm.noVoxels)]

# Find the distribution of the t-statistic for each permutation

permutation.dist <- matrix(0, n, perm.noVoxels)

for(i in 1:n){

data.temp <- perm.mat[,i]*sub.dataMatrix

ttest.out <- apply(data.temp, 2,

function(x) {

temp <- t.test(x)

temp$statistic

})

permutation.dist[i,] <- ttest.out

}

**Figure 7:**
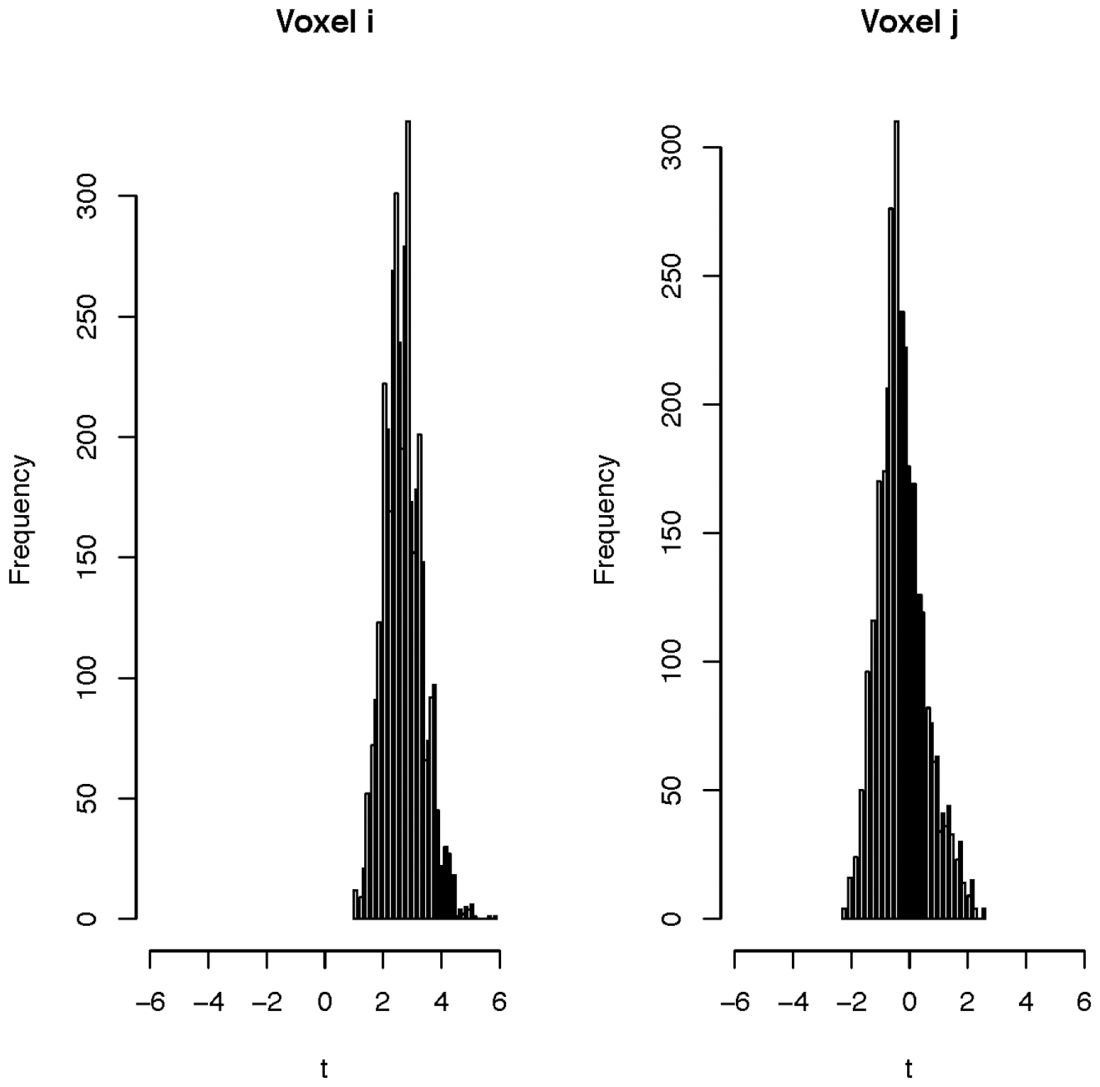
Permutation histograms of t-statistics for two voxels.

The permutation distributions of the t-statistics for the two randomly selected voxels are shown in Figure 7. Using the permutation distribution, one can compute the p-value of observing the true labeling of disease status and make a decision about whether to reject the null hypothesis or not.

### 7 Connectivity analysis

Functional connectivity refers to the analysis of correlations of measured brain function between potentially remote areas of the brain [15]. In fMRI, this translates to evaluating correlations, or perhaps other forms of associations between voxels or regions. We emphasize that fMRI represents only one modality to investigate functional connectivity. Others include: EEG, MEG and PET. When investigating connectivity, it is important to consider the goals of the analysis and the spatial/temporal resolution of the technology under study [15]. Such analyses have become so influential in fMRI, that they have been abbreviated as fc-fMRI (for functional connectivity fMRI). A subset of such analyses considers functional connectivity while a subject is at rest in the scanner, and is often referred to as resting state functional connectivity (rs-fc-fMRI). Much of the analysis of rs-fc-fMRI centers around the so-called “default mode network”, a hypothesized brain network that is activated in subjects at rest [44].

#### 7.1 Connectivity and preprocessing

The analysis of brain connectivity data requires special preprocessing that may be useful for paradigm-related studies, but is crucial for the study of connectivity. Most notably it is addressing background nuisance signals. These include signals related to cardiac function and respiration. Because one is concerned with correlations between voxels, such background effects can create spurious relationships that are not of interest. In contrast, they are less crucial in a paradigm-related study, since the paradigm is likely not aliased with the nuisance signal, though to the contrary, they are probably not orthogonal to the paradigm. In event-related designs where events are presented randomly, the randomization mechanism helps ensure a lack of relationship with nuisance signals.

There are several methods for addressing nuisance signals in fc-fMRI. These include a connectivity analysis of the ventricles, the spaces in the brain filled with cerebrospinal fluid. There should be no measured connectivity of interest in the ventricles, hence any correlations found here and elsewhere likely represent nuisance signals. A similar approach would look at white matter, though such analyses would be highly dependent on very accurate registration. These issues should be taken into account when preprocessing the data.

#### 7.2 Seed voxel approaches

Assuming that there are no nuisance signals in the brain, the correlations of the fMRI time courses can be computed for each pair of voxels. The number of these pairs is often very large and the brute force computation of the correlations may be computationally expensive. Instead, one can analyze regions of interest (ROI), which can be obtained, for example, by using the anatomy of the brain or by independent component analysis as described in Section 7.3. For each ROI, a seed voxel can be chosen as one of the voxels in the ROI or as the average time course of all voxels in the ROI. The correlation map of the seed voxel with the remaining voxels in the brain is called the connectivity map. The obtained connectivity maps may be used, for instance, to compare the connectivity of an ROI between subjects in two disease groups.

Generally, after calculating the raw correlation values, a Fisher R to Z transform is performed before analyzing the correlation map. The transform is defined as
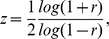
where r is a correlation value. The resulting connectivity maps should have approximately normally distributed values. Standardizing this matrix by the standard error 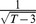, where T is the number of time points used to compute the correlation, is frequently used, especially when different participants have different lengths of scans. These maps then can be analyzed using random Markov field corrections for multiple comparisons, using t-tests or the nonparametric rank-sum test, or other methods for testing differences across groups [15].

#### 7.3 Singular value decomposition and independent component analysis

Since the fMRI data are very large and complex, dimension reduction techniques are often used to identify important signals in the data as well as help with visualization. For example, singular value decomposition, a well-known statistical dimension reduction technique, is often used for preprocessing the data before testing or inference.

Another commonly used dimension reduction method is independent component analysis (ICA, see [45] for an extensive overview). It is mainly used in fMRI to obtain functional networks in the brain. It has been implemented both for resting-state and task-related fMRI experiments. Here we show the use of ICA for an attention task using one of the SPM example datasets [46]. Four different conditions were explored: fixation, attention, no attention and stationary.

There are several methods one can use for applying ICA. For instance, the R package AnalyzeFMRI has a function f.ica.fmri that is based on the fastICA algorithm [45]. If the data are in Analyze format, where each 3D scan is saved in a separate file, the individual scans per time point have to be combined into one 4D image in order to use f.ica.fmri. Here, the 4D image is saved as fullimg.img, where one of the dimensions is time and the other three are the image dimensions.

library(AnalyzeFMRI)

# Specify the number of components that should be estimated

m<-10

f<- f.ica.fmri(“fullimg.img”, n.comp  = m)

The fastICA package in R can be used directly for ICA analysis as follows. We create a matrix dataMatrix that is a 2D (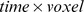) version of the fMRI data as described in Section 4.2, and apply the fastICA function to obtain 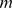 independent components.

library(fastICA)

f<- fastICA(t(dataMatrix), n.comp  = m)

The variable 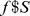 contains the collection of the independent components. Next, we back-reconstruct each of the components as an image. The networks are identified as the regions that are highly activated in the corresponding independent component. In other words, to obtain the brain networks we can threshold the values of each column in 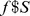. We will use a simple thresholding tool here by including the values greater than a threshold 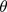. We also save the arrays in image format for visualization. Note, we assume imageDim constains the image dimensions as computed in Section 4.2.

theta <- 2

S <- f$S

S[S < =  theta] <- 0

imageDimComp <- c(imageDim, m)

img <- array( 0, dim  =  imageDimComp)

for(i in 1:m){

temp2 <- array(0, imageDim)

temp2[mask] <- S[,i]

img[,,,i] <- temp2[,,]

}

f.write.analyze(img,

file  =  “fica”,

size  =  “float”,

pixdim  =  c(3, 3, 3, 1, 0, 0, 0),

originator  =  c(27, 38, 18, 0, 0))

**Figure 8:**
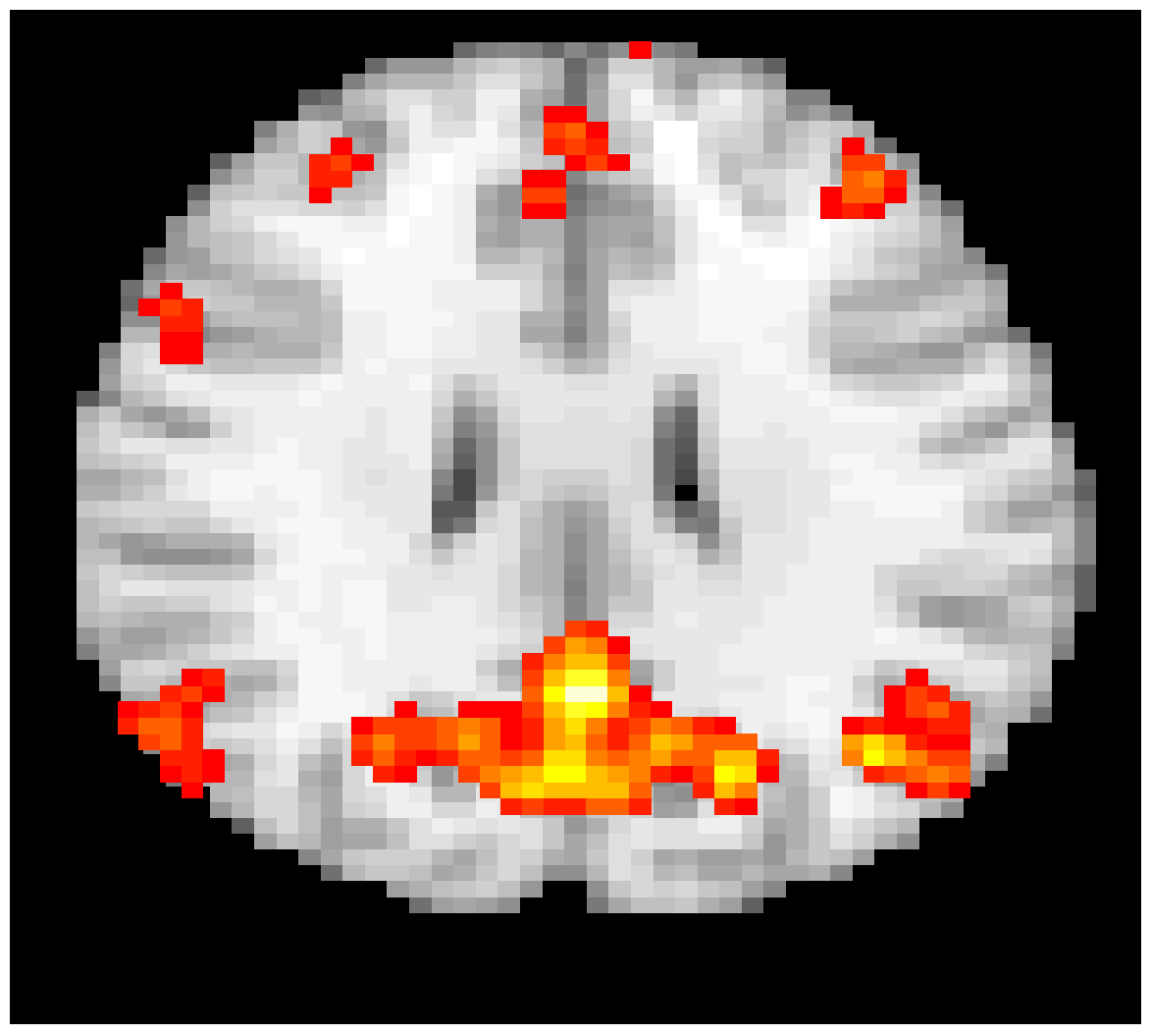
The default brain network obtained via fastICA and overlaid on a template brain.

Figure 8 shows a slice of the default brain network obtained by the above fastICA algorithm thresholded and overlaid on a template brain.

#### 7.4 Group ICA

Group ICA is the extension of the ICA method used to obtain brain networks from fMRI datasets in a population. The method was first introduced by [47] and has since been widely used in the neuroimaging literature [48], [49]. The method is based on concatenating the two-dimensional 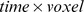 matrices observed for each subject and applying principal component analysis to reduce the dimension to the desired number of components. Then the single subject ICA algorithm is applied to the resulting matrix to obtain the common independent components for the group.

There are two different choices for matrix concatenation: one assumes that the spatial brain networks are statistically independent (spatial group ICA), the other assumes that the time courses are statistically independent (temporal group ICA). Spatial group ICA, which assumes common spatial networks across subjects yet different temporal mixing matrices is more frequently used. By assuming common spatial maps, one can concatenate all subjects' data in the temporal domain, and apply ICA to the aggregated data matrix. Below is an example for spatial group ICA:

fileDir <- “pathToImageFile”

files <- dir(fileDir, pattern  =  “*.img”, full.names =  TRUE)

groupDataMatrix <- NULL

for (file in files) {

img <- f.read.analyze.volume(file)

imageDataMatrix <- t(apply(img, 4, function(imgi) imgi[mask]))

rmeans <- rowMeans(imageDataMatrix)

cmeans <- colMeans(imageDataMatrix)

imageDataMatrix <- sweep(imageDataMatrix, 2, cmeans, “-”)

imageDataMatrix <- sweep(imageDataMatrix, 1, rmeans, “-”)

groupDataMatrix <- rbind(groupDataMatrix, imageDataMatrix)

}

m <- 20

f <- fastICA(t(groupDataMatrix), n.comp  =  m)

### 8 Displaying results

Different modalities can be used for displaying the results obtained by the analysis of fMRI data. In most cases, a group of voxels in the brain is found by the analyses and can be visualized by overlaying the voxels on a template brain. For instance, in the case of ICA, the functional networks obtained can be displayed by overlaying the networks on a template brain. In this section, we will discuss only a few of the functions that can be used for visualization in R or other software.

#### 8.1 Within R

The function image() in R can be used for plotting the 2D images for each value of the third dimension to display the images for each slice of the brain. The rgl and misc3d packages have tools for creating 3D renderings and isosurfaces directly in R. We have found that images generally need to be slightly downsampled to avoid memory issues. Usually, this does not impact the appearance of the graphics dramatically. The misc3d package has some functions for plotting images and rendering in 3D activation maps for fMRI (http://www.tandfonline.com/doi/abs/10.1198/jcgs.2010.191ed). Contour3d and image3d are useful functions for displaying results and embedding 3D objects into PDFs or RGL objects.

When plotting images in R, a few important points should be taken into consideration. When overlaying selected voxels on a template brain, note that the template brain and the statistical image results: 1) may not have the same array dimensions, 2) voxels may not correspond to the same real world dimensions, such as the voxels in the template being 1 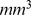 while the voxels in the statistical image being 3 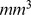, 3) the images may not have the same origin. This information is contained in the raw and template image's header files. The origin is the point that is used to match images. Hence, when overlaying images if one appears shifted, it is probably due to incorrectly set values of the image origins. Another problem that can occur is that the voxels are not square. For example, the 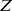 physical dimension (in the direction from the feet to the head), is often different than that of the other two. If this is not accounted for, the images will appear strange, not unlike a geographical map with an incorrect contrast ratio. These issues can all be handled in R.

Finally, when plotting the image via the image function, the number of colors used in plotting is 12 by default. That number can be increased by changing the parameter col (e.g., col  =  heat.colors(100)) to obtain a smoother image. In addition, it is common to plot fMRI data in greyscale as shown in Figure 2, hence the color spectrum can be changed to col  =  grey.colors(100) in the image function to achieve that.

#### 8.2 Third party software

There are several visualization software platforms that can be used for examining fMRI data. They vary from software for quick visualization to 3D renderings of the brain with complicated visualizations of slices and cross-sections. For instance, 3D Slicer and Mipav can be used to obtain 3D renderings of the brain overlaid on template brains. These software platforms can also be used for masking the brain or cropping regions of interest as well as registration. The videos at http://www.youtube.com/watch?v=GNsWRnm7gQw show a step-by-step illustration of 3D Slicer or Mipav for visualization and analyses.

## Further reading

Below we give an incomplete list of papers in the area.

• A good starting point for fMRI is the SPM book [15]
• Nicole Lazar's introduction to fMRI [50] and book [18]
• Martin Lindquist's manuscript on fMRI [17]
• Thomas Nichol's overview paper on permutation testing in fMRI [43]
• Vincent Calhoun's introductions to ICA and group ICA in fMRI [51], [47]
• Jonathan Taylor's manuscript about the HRF [52]
• Technical papers about random field theory are [53], [54]
• An accessible discussion including permutation testing can be found in [55] and [56]
• A somewhat dated but still very relevant, overview of the analysis of fMRI data [57]
• A more recent tutorial can be found here [58]
• An expository article on R and neuroimaging [59]
• Our work on singular value decomposition for fMRI [60]

This manuscript gives an admittedly very brief introduction and quick start guide for fMRI. For those interested in pursuing further development in the area, we recommend investigating processing pipelines as a next important step.
